# Prenatal Polycyclic Aromatic Hydrocarbon (PAH) Exposure and Child Behavior at Age 6–7 Years

**DOI:** 10.1289/ehp.1104315

**Published:** 2012-03-14

**Authors:** Frederica P. Perera, Deliang Tang, Shuang Wang, Julia Vishnevetsky, Bingzhi Zhang, Diurka Diaz, David Camann, Virginia Rauh

**Affiliations:** 1 Department of Environmental Health Sciences, Mailman School of Public Health, Columbia University, New York, New York, USA; 2 Columbia Center for Children’s Environmental Health, Columbia University, New York, New York, USA; 3 Department of Biostatistics, Mailman School of Public Health, Columbia University, New York, New York, USA; 4 Southwest Research Institute, San Antonio, Texas, USA; 5 The Heilbrunn Department of Population and Family Health, Columbia University, New York, New York, USA

**Keywords:** air pollution, child behavior, PAH, prenatal

## Abstract

Background: Airborne polycyclic aromatic hydrocarbons (PAH) are widespread urban air pollutants from fossil fuel burning and other combustion sources. We previously reported that a broad spectrum of combustion-related DNA adducts in cord blood was associated with attention problems at 6–7 years of age in the Columbia Center for Children’s Environmental Health (CCCEH) longitudinal cohort study.

Objectives: We evaluated the relationship between behavioral problems and two different measures of prenatal exposure—both specific to PAH—in the same cohort.

Methods: Children of nonsmoking African-American and Dominican women in New York City (NYC) were followed from *in utero* to 6–7 years. Prenatal PAH exposure was estimated by personal air monitoring of the mothers during pregnancy as well as by the measurement of DNA adducts specific to benzo[*a*]pyrene (BaP), a representative PAH, in maternal and cord blood. At 6–7 years of age, child behavior was assessed using the Child Behavior Checklist (CBCL) (*n* = 253). Generalized linear models were used to test the association between prenatal PAH exposure and behavioral outcomes.

Results: In multivariate analyses, high prenatal PAH exposure, whether characterized by personal air monitoring (greater than the median of 2.27 ng/m^3^) or maternal and cord adducts (detectable or higher), was positively associated with symptoms of Anxious/Depressed and Attention Problems (*p* ≤ 0.05).

Conclusion: These results provide additional evidence that environmental levels of PAH encountered in NYC air can adversely affect child behavior.

Polycyclic aromatic hydrocarbons (PAH) such as benzo[*a*]pyrene (BaP) are released to the air during incomplete combustion of fossil fuel, tobacco, and other organic material (Bostrom et al. 2002). In New York City (NYC) and other urban areas, traffic and residential heating are major sources. Urban, minority populations in the United States often have disproportionate exposure to air pollution and are at greater risk for adverse health and developmental outcomes (Olden and Poje 1995; Perera et al. 2002). Illustrating widespread exposure to these pollutants, 100% of the mothers in the Columbia Center for Children’s Environmental Health (CCCEH) NYC cohort had detectable levels of PAH in prenatal personal air samples. In addition, 40% reported environmental tobacco smoke (ETS) exposure during pregnancy (Perera et al. 2003).

Because of the heightened susceptibility of the fetus and infant, exposures to PAH and other environmental pollutants during the prenatal and early postnatal stages are of particular concern (Anderson et al. 2000; Grandjean and Landrigan 2006; National Research Council 1993; Perera et al. 2004; World Health Organization 1986). In particular, the prenatal period is thought to be highly sensitive to neurotoxic effects of environmental contaminants (Nijland et al. 2008; Rodier 2004). Laboratory experiments have indicated that the fetal brain and nervous system may be particularly sensitive to PAH (Brown et al. 2007; McCallister et al. 2008; Wormley et al. 2004). PAH are transferred across the placenta and the fetal blood brain barrier [reviewed by Brown et al. (2007)]. Following gestational exposure in humans, DNA adducts formed by BaP and other PAH have been detected in maternal and cord blood samples in a range of populations (Perera et al. 2005). PAH exposure has also been linked to epigenetic effects (Perera et al. 2009b). In studies in laboratory animals, BaP exposure also has been associated with AhR (aryl hydrocarbon receptor) up-regulation in gestationally exposed rats, suggesting possible endocrine disruption (Wu et al. 2003).

In the CCCEH NYC cohort, prenatal exposure to PAH has previously been associated with multiple adverse effects including developmental delay at 3 years of age (Perera et al. 2006) and reduced IQ at 5 years of age (Perera et al. 2009a). Experimental studies exposing laboratory animals to PAH during the prenatal and neonatal periods have reported neurodevelopmental and behavioral effects including impairment of memory and ability to learn (Brown et al. 2007; Wormley et al. 2004), anxiety, and depression-like symptoms in the absence of overt toxicologic effects (Saunders et al. 2002, 2003, 2006; Takeda et al. 2004; Wormley et al. 2004; Yokota et al. 2009). Anxiety and depression are internalizing problems that can affect learning (Emslie 2008; Wood 2006).

We have previously found that a wide spectrum of bulky hydrophobic DNA adducts, including those formed by PAH, nitro-PAH, and aromatic amines, detected by the ^32^P-postlabeling assay in umbilical cord blood from cohort children were associated with symptoms of anxiety/depression and attention problems during childhood (Perera et al. 2011). Here, in addition to prenatal PAH air monitoring, we have used a chemical-specific biomarker of exposure (BaP–DNA adducts) to examine the relationship between prenatal PAH exposure and child behavior. The BaP–DNA adducts are specific to a representative member of the PAH class and therefore more directly complement the prenatal air monitoring of BaP and other PAH than the ^32^P-radiolabeled adducts, which represent an array of pollutants in addition to PAH.

We have examined children’s behavior at 6–7 years of age in relation to prenatal exposure to PAH and BaP–DNA adducts using the Child Behavior Checklist (CBCL) (Achenbach and Rescorla 2001). We focused on the syndromes and problems of *a priori* interest (anxiety/depression and attention problems) based on the experimental findings for PAH (Saunders et al. 2002, 2003, 2006; Takeda et al. 2004; Wormley et al. 2004; Yokota et al. 2009). This is the first report of associations between child attentional and behavioral problems, on the one hand, and two complementary specific measures of prenatal PAH exposure on the other: monitored air concentrations of PAH and a PAH-specific biomarker of exposure.

## Methods

*Sample selection.* A complete description of the NYC cohort and study design appears elsewhere (Perera et al. 2003, 2006). Briefly, African-American and Dominican women who resided in Washington Heights, Harlem, or the South Bronx in New York City (USA) were recruited between 1998 and 2003 through the local prenatal care clinics into a prospective cohort study. To reduce the potential for confounding, enrollment was restricted to women who were nonactive cigarette smokers in the age range of 18–35 years; nonusers of other tobacco products or illicit drugs; free of diabetes, hypertension, or known HIV; and had initiated prenatal care by the 20th week of pregnancy. Postnatal interviews were administered to the mothers in person when the child was 6 months of age and annually thereafter to determine changes in children’s health, residence, and ETS exposure. Developmental status was assessed every 1–2 years. The Institutional Review Board of the New York Presbyterian Medical Center approved the study; the mothers provided informed consent for themselves and their children < 7 years of age, and children ≥ 7 years provided assent.

A total of 617 mother–child pairs had complete prenatal PAH monitoring and prenatal questionnaire data. By the time of this analysis, 431 children had reached 7 years of age, and 294 children had the CBCL completed at age 6–7. The sample included in the present analysis was composed of 253 of those children who also had available data on explanatory or potential confounding variables of interest. Table 1 compares characteristics of this sample with those subjects who were not included due to missing covariates or CBCL data (*n* = 364). The two groups were similar with respect to level of PAH and ETS exposure, gestational age, maternal education, and maternal demoralization, as measured by the PERI (Psychiatric Epidemiology Research Interview) Demoralization Scale (Dohrenwend et al. 1978); however, they differed with respect to the home caretaking environment as measured by the HOME (Home Observation for Measurement of the Environment) Inventory (Bradley 1994) (*p* = 0.03), sex of the child (*p* = 0.05), and ethnicity (*p* < 0.0001). Specifically, the group included in the analysis had a higher proportion of girls and African Americans and a more favorable home environment.

**Table 1:** Characteristics of sample.

Subjects in the analysis (n = 253)	Subjects not includeda (n = 364)		
Variable	Mean ± SD or %	n	Mean ± SD or %
PAH (ng/m3)		3.23 ± 2.76		364		3.13 ± 4.10
Prenatal ETS exposure (% yes)		37.55		364		32.14
Percent femaleb		56.52		364		48.35
Gestational age (weeks)		39.51 ± 1.57		364		39.59 ± 1.35
Maternal demoralization scorec		1.15 ± 0.62		324		1.15 ± 0.65
Maternal education (years)		11.80 ± 2.04		364		11.85 ± 2.31
Maternal TONI-2 scored		20.87 ± 8.96		232		20.57 ± 8.62
Home environmentb,e		39.87 ± 5.66		215		38.60 ± 6.82
Ethnicity (% African American)b,f		49.04		364		30.22
Heating season (% yes)g		56.92		362		58.01
Change of residence before the age of testing		64.03		67		64.18
aSome subjects were not included due to missing CBCL or information on at least one covariate. bSignificantly different between the two groups based on Pearson’s chi-square test. cMaternal demoralization during pregnancy. dNonverbal intelligence measured by the TONI-2. eHOME Inventory as a measure of the home caretaking environment. fPercent African American; the rest are Dominican. gThird trimester in heating season.						

*Personal interview.* A 45-min questionnaire was administered by a trained bilingual interviewer during the last trimester of pregnancy to obtain demographic information, residential history, and health and environmental data such as active smoking (to confirm nonactive smoking status) and passive smoking. In this cohort, self-reported exposure to ETS was correlated with cotinine measured in cord blood (*r* = 0.44, *p* < 0.0001). The questionnaire also elicited information on dietary PAH (consumption of broiled, fried, grilled, or smoked meat) and socioeconomic information related to income and education (Perera et al. 2003). Postnatal interviews were administered in person when the child was 6 months old and annually thereafter to determine changes in residence, exposure to ETS, and health and environmental conditions. The quality of the proximal caretaking environment was assessed at child’s age 3 years using Caldwell and Bradley’s HOME Inventory (Bradley 1994).

*Prenatal personal PAH assessment.* Personal monitoring was carried out during the third trimester of pregnancy as previously described (Perera et al. 2003). Vapors and particles of ≤ 2.5 µm in diameter were collected on a precleaned quartz microfiber filter and a precleaned polyurethane foam (PUF) cartridge backup (University Research Glassware, Chapel Hill, NC). The samples were analyzed at Southwest Research Institute (SWRI) for BaP, benz[*a*]anthracene, chrysene, benzo[*b*]fluroanthene, benzo[*k*]fluroanthene, indeno[1,2,3-*cd*]pyrene, dibenz[*a,h*]anthracene, and benzo[*g,h,i*]perylene. For quality control (QC), each personal monitoring result was assessed for accuracy in flow rate, time, and completeness of documentation. Samples not meeting QC criteria for adequacy were not included in the analysis.

We validated 48-hr personal monitoring as an indicator of longer-term, integrated exposure in two ways. In a subset of the NYC cohort (*n* = 84), indoor air was monitored over 6 weeks during the third trimester concurrent with the personal air monitoring: The prenatal personal air concentrations were significantly correlated with indoor levels of PAH (sum of the 8 PAH) (*r* = 0.58, *p-*value < 0.001) (Rundle et al. 2012). In addition, among a subset of pregnant women (*n* = 80) participating in a parallel birth cohort study in Poland and simultaneously monitored for personal, indoor, and outdoor airborne PAH, all three measurements were found to be highly correlated (pair-wise Spearman’s coefficients ≥ 0.84, *p* < 0.01) (Choi et al. 2008), supporting the use of personal monitoring to integrate indoor and outdoor exposure. A caveat is that the air concentrations of PAH in the Polish study were 10 times higher than in the NYC cohort (Jedrychowski et al. 2005).

*Biomarkers.* Adducts in white blood cells reflect individual variation in exposure, absorption, metabolic activation, and DNA repair, providing a biological dosimeter and marker of potential risk. We collected umbilical cord blood at delivery and maternal blood (30–35 mL) generally on the first day after delivery and transported the samples to the CCCEH Molecular Epidemiology Laboratory within several hours of collection. The buffy coat, packed red blood cells, and plasma were separated and stored at –70°C. BaP–DNA adducts in extracted white blood cell DNA were analyzed using the high performance liquid chromatography (HPLC)/fluorescence method of Alexandrov et al. (1992), which detects BaP tetraols. The method has been described previously (Perera et al. 2004).

*Behavioral outcomes.* The CBCL is a widely used instrument shown to be sensitive to diverse prenatal environmental exposures, including stress events, smoking during pregnancy, and exposure to various pollutants (Axtell et al. 2000; Rauh et al. 2006; Robinson et al. 2008; Wasserman et al. 2001). Research workers trained in neurodevelopmental testing administered the 118-item CBCL for children 6–18 years of age (Achenbach and Rescorla 2001) to the mothers in English or Spanish. Specifically, the mothers completed the CBCL with guidance as needed from the research workers. The syndrome scores were computed for the two *a priori* domains of interest (Anxious/Depressed and Attention Problems) by summing the scores on the specific items, yielding a continuous raw score. The raw scores were also converted to standardized *T*-scores, generated according to the procedure of Abramowitz and Stegun (1968). The *T*-score is truncated (Petersen et al. 1989): A score of 50 is assigned to those with percentiles of raw scores ≤ 50 based on a reference population (Achenbach and Rescorla 2001), whereas children with raw score percentiles > 50 are assigned an actual *T*-score.

The CBCL also yields scales derived from the *Diagnostic and Statistical Manual of Mental Disorders, 4th ed.* (DSM-IV; American Psychiatric Association 2000) that are intended to approximate clinical diagnoses. The DSM-IV scores are dichotomized using a borderline or clinical cut point corresponding to the 93rd percentile in a reference population for each domain (Achenbach and Rescorla 2001). Children were thus classified as in the borderline or clinical range (*T-*score ≥ 65) or in the normal range (*T-*score < 65) for the DSM-IV–oriented Anxiety Problems and for Attention Deficit/Hyperactivity Problems.

Because maternal intelligence is a known predictor of child neurodevelopment across populations, the *Test of Nonverbal Intelligence, 2nd ed.* (TONI-2) (Brown et al. 1990) was administered to the mothers when the child was about 3 years of age. The TONI-2 is a 15-min, language-free measure of general intelligence, relatively stable and free of cultural bias. The quality of the proximal caretaking environment is also a predictor of child neurodevelopment, so Caldwell and Bradley’s HOME Inventory (Bradley 1994) was administered at about 3 years to assess physical and interactive home characteristics. Because ambient PAH concentrations are higher in the winter months (heating season), we adjusted for season of monitoring (heating vs. nonheating season).

*Statistical analysis.* As in prior analyses (Perera et al. 2003), a composite PAH variable was computed as the sum of the eight intercorrelated PAH air concentration measures (*r*-values ranging from 0.38–0.96; all *p* < 0.001 by Pearson’s correlation). This variable was dichotomized at the median for the parent population (2.273 ng/m^3^) to obtain a measure of high/low exposure. In separate analyses, PAH were log transformed and treated as a continuous variable. BaP–DNA adduct data were available for 223 maternal samples and 148 cord blood samples. Adduct levels were dichotomized as detectable/nondetectable, with detectable levels found in 87 maternal and 56 cord blood samples.

Covariates were selected based on whether they were significant contributors to the model (at *p* ≤ 0.1) for at least one of the outcomes. They included maternal self-report of ETS exposure during pregnancy, sex of child, gestational age of the child, mother’s intelligence, mother’s completed years of education before birth of the child, maternal prenatal demoralization, child’s age at assessment, the quality of the early home caretaking environment assessed when the child was around 3 years of age, and season at time of monitoring (heating vs. nonheating) (Table 1). Dietary PAH was not a predictor of outcomes at a *p* ≤ 0.1 and was not included in the model. Gestational age was based on medical record data for almost all subjects. To adjust for postnatal exposure to PAH, further analyses included change of residence before the age of testing as a proxy for possible change in PAH exposure or PAH metabolites measured in child urine collected at 3 years of age. We also adjusted for maternal reported postnatal ETS exposure before the age of testing. Correlations between continuous airborne PAH and ETS and dietary PAH, respectively, were not significant using Spearman rank-order correlation and Pearson correlation (*r* = –0.01, *p* = 0.82 and *r* = 0.09, *p* = 0.18, respectively; *n* = 238 for both). The correlation between PAH and cord lead levels, measured at the Centers for Disease Control and Prevention (CDC) using inductively coupled plasma mass spectrometry (CDC/Division of Laboratory Science 2003), was not significant using Pearson correlation (*r* = –0.01, *p* = 0.77, *n* = 168) in the limited subset with both measurements.

We used continuous raw scores and dichotomized *T*-scores in the analyses of the CBCL syndrome scores. We applied the Poisson model on the raw syndrome scores as they are counts data with a right skew. The syndrome *T*-scores were dichotomized at 65 (the cutoff for the borderline and clinical range) (Achenbach and Rescorla 2001) and analyzed using a logistic model. Similarly, we used logistic regression to analyze the dichotomized DSM-IV–oriented Anxiety Problems and Attention Deficit/Hyperactivity Problems.

This analysis involved multiple comparisons. Although the Bonferroni method can be overly conservative (Westfall and Young 1993), we report both the uncorrected and corrected *p-*values. The Bonferroni-adjusted significance level was α = 0.05/6 = 0.0083 (six outcomes tested).

## Results

*Using personal air monitoring data as the exposure measure.* Table 2 provides the distribution of CBCL scores, while Table 3 shows the distribution of children in the normal and borderline or clinical range for logistic models. In simple Poisson regression (without adjustment for covariates), the children of women with higher monitored prenatal exposure to PAH had significantly higher scores, and thus more symptoms of Anxious/Depressed and Attention Problems at 6–7 years than did children with lower prenatal PAH exposure. In the full Poisson model after adjusting for possible confounders, higher monitored PAH exposure was associated with a significantly higher symptom score of Anxious/Depressed (1.45 times that of the low PAH exposure group, 95% confidence interval (CI): 1.22, 1.72; *p* < 0.0001) and with the symptom score of Attention Problems (1.28 times that of the low PAH exposure group; 95% CI: 1.10, 1.48; *p* = 0.001, after adjustment using the Bonferroni method) (Table 4).

**Table 2:** Distribution of outcomes in n = 253 children with PAH measurements.

Outcome	Score range			Mean of scores			Percent in borderline or clinical rangeb
	T-scorea	Raw score	T-score	Raw score			
Anxious/depressed		50–82		0–17		55.54		2.55		6.32
Attention problems		50–83		0–16		56.19		3.34		6.72
Anxiety problems (DSM-IV)				0–9				1.53		9.48
Attention deficit/hyperactivity problems (DSM-IV)				0–14				3.28		7.91
aThe T-score is truncated (Petersen et al. 1989); that is, a score of 50 is assigned to those with percentiles of raw scores ≤ 50 based on a reference population (Achenbach and Rescorla 2001). bThe syndrome T-scores were dichotomized at 65 as the cutoff for the borderline and clinical range; the DSM‑IV–oriented Anxiety Problems scale was also dichotomized at 93rd percentile.										

**Table 3:** Distribution of dichotomized CBCL outcomes for logistic models.

Anxious/depressed			Attention problems			DSM-IV Anxiety Problems			DSM-IV Attention Deficit/Hyperactivity Problems		
Normal range	Borderline or clinical	Normal range	Borderline or clinical	Normal range	Borderline or clinical	Normal range	Borderline or clinical	Total
PAH		237		16a		236		17		229		24		233		20		253
Maternal adducts		212		11		208		15		204		19		205		18		223
Cord adducts		136		12		137		11a		130		18		137		11		148
aFewer than 5 children were in the borderline or clinical range in the low-exposure group, making these results less reliable.																		

**Table 4:** Associations between all measures (PAH exposure, maternal and cord PAH (BaP)–DNA adducts measured via HPLC, and cord PAH (BaP)–DNA adducts measured via 32P) and CBCL Syndrome and DSM Oriented outcomes in children ages 6–7 years.^a^

Syndrome scale Anxious/Depressed											Syndrome scale Attention Problems										
Poisson Raw					Logistic dichotomized T					Poisson Raw					Logistic dichotomized T				
Exposure	Exp beta	95% CI	p-Value	OR	95% CI	p-Value	Exp beta	95% CI	p-Value	OR	95% CI	p-Value
PAH (high/low) (n = 253, n = 16)		1.45		(1.22, 1.72)		< 0.0001		8.89		(1.70, 46.51)		0.010b		1.28		(1.10, 1.48)		0.001		3.79		(1.14, 12.66)		0.030
Maternal HPLC adductsc (n = 223)		1.23		(1.04, 1.46)		0.019		1.42		(0.38, 5.35)		0.603		1.25		(1.08, 1.45)		0.003		2.24		(0.74, 6.77)		0.153
Cord HPLC adductsc (n = 148)		1.46		(1.19, 1.78)		< 0.001		2.56		(0.69, 9.43)		0.159		1.32		(1.11, 1.58)		0.002		4.06		(0.99, 16.63)		0.051b
Cord 32P adductsd,e (n = 205)		–0.03		(–0.22, 0.16)		0.773		1.42		(0.45, 4.46)		0.544		0.22		(0.06, 0.38)		0.009		3.30		(1.22, 12.54)		0.022

In logistic regression on the syndrome scales, higher PAH exposure was significantly associated with both Anxious/Depressed and Attention Problems (Table 4). However, because < 5 children were in the borderline or clinical range in the low-exposure group for Anxious/Depressed, these results are less reliable.

In logistic regression after adjustment, higher PAH exposure was associated with higher odds of DSM-IV–oriented Anxiety Problems [odds ratio (OR) = 4.59; 95% CI: 1.46, 14.27; *p-*value = 0.009] (Table 4). In contrast, high PAH exposure was not significantly associated with DSM-IV–oriented Attention Deficit/Hyperactivity Problems (OR = 2.30; 95% CI: 0.79, 6.70).

In separate models with log-transformed continuous PAH as the independent variable, monitored PAH exposure was positively associated with the syndrome score of Anxious/Depressed (1.20 times that of the low PAH exposure group; 95% CI: 1.06, 1.37; *p* = 0.004). Log-transformed PAH exposure was also positively associated with DSM-IV–oriented anxiety (OR = 2.03 for a 1-unit increase in log PAH; 95% CI: 0.97, 4.26; *p* = 0.060) and with Attention Problems (OR = 1.54; 95% CI: 0.70, 3.40; *p* = 0.283).

Prenatal ETS was a significant predictor of Anxious/Depressed and Attention Problems at 6–8 years of age. Maternal prenatal demoralization was also a significant predictor of most of the outcomes evaluated. Using change of residence before the age of testing as a proxy for variation in PAH exposure between the pre- and postnatal periods, the significance of the associations between higher prenatal PAH and outcomes was materially unchanged, both for symptoms of Anxious/Depressed (1.45 times that of the low PAH exposure group; 95% CI: 1.22, 1.72; *p* < 0.0001) and for DSM-IV–oriented Anxiety Problems (OR = 4.36; 95% CI: 1.41, 13.44; *p* = 0.011). The same was true for Attention Problems (1.27 times that of the low PAH exposure group; 95% CI: 1.10, 1.47; *p* = 0.001). After controlling for urinary PAH metabolites at 3 years of age in the subset with available biomarker data (*n* = 191), the association between prenatal PAH and syndrome scores became stronger (Anxious/Depressed: 1.72 times that of the low PAH exposure group; 95% CI: 1.40, 2.10; *p* = < 0.0001; Attention Problems: 1.38 times that of the low PAH exposure group; 95% CI: 1.16, 1.64; *p* = 0.0002) [see Supplemental Material, Table 4 (http://dx.doi.org/10.1289/ehp.1104315)]. Controlling for postnatal exposure to ETS before age of testing did not materially influence the results (data not shown).

In separate analyses, we adjusted for ETS exposure using log-transformed cord cotinine as a continuous variable in a subset of the sample with available data (*n* = 194). Associations with PAH were similar to those adjusted for self-reported ETS exposure (Anxious/Depressed: 1.40 times that of the low PAH exposure group; 95% CI: 1.15, 1.71; *p* = 0.001; Attention Problems: 1.24 times that of the low PAH exposure group; 95% CI: 1.04, 1.47; *p* = 0.016). The odds ratio for DSM-IV–oriented Anxiety Problems was closer to the null, though still positive (OR = 2.9; 95% CI: 0.81, 10.43; *p* = 0.1); this may be attributable to reduced sample size.

In separate models, we further controlled for other neurotoxic environmental exposures measured in the CCCEH cohort including bisphenol A (BPA), the pesticide chlorpyrifos, and phthalates using a summary score for the number of these coexposures that were above the median level for the cohort. The associations between PAH and CBCL Anxious/Depressed and Attention Problems syndrome scores remained similar when adjusting for the summary scores of these other pollutants; however, the sample size was reduced to *n* = 110 (Anxious/Depressed: 1.63 times that of the low PAH exposure group; 95% CI: 1.22, 2.17; *p* = 0.001; Attention Problems: 1.54 times that of the low PAH exposure group; 95% CI: 1.22, 1.94; *p* = 0.0002; DSM-IV–oriented Anxiety Problems: OR = 7.19; 95% CI: 0.88, 58.48; *p* = 0.065; DSM-IV–oriented ADHD Problems: OR = 4.77; 95% CI: 0.74, 30.58; *p* = 0.100). The summary score was not a significant independent predictor of any of the outcomes.

*Using PAH (BaP)–DNA adducts as the exposure measure.* We analyzed the relationship between cord and maternal BaP–DNA adducts and CBCL outcomes, using the same approach as for monitored PAH, with adjustment for the same covariates.

As shown in Table 4, in the Poisson model detectable levels of PAH adducts in both maternal blood and cord blood were associated with significantly higher scores on the CBCL syndromes of Anxious/Depressed (1.23 times that of the nondetectable adducts group; 95% CI: 1.04, 1.46; *p* = 0.019 for maternal; and 1.46 times that of the nondetectable adducts group; 95% CI: 1.19, 1.78; *p* < 0.001 for cord). The same was true for Attention Problems (1.25 times that of the nondetectable adducts group; 95% CI: 1.08, 1.45; *p* = 0.003 for maternal; and 1.32 times that of the nondetectable adducts group; 95% CI: 1.11, 1.58; *p* = 0.002 for cord). Thus, the two measures (personal monitoring and PAH (BaP)-specific DNA adducts) gave similar results for the CBCL symptom scores in the Poisson model. In logistic regression on the dichotomized syndrome scales, although detectable levels of cord adducts were associated with Attention Problems, < 5 children were in the borderline or clinical range in the low-exposure group, making these results less reliable.

Similar to monitored PAH, cord and maternal adducts were not associated with DSM-IV–oriented Attention Deficit/Hyperactivity Problems. In contrast to monitored PAH, logistic regression did not show significant associations between BaP–DNA adducts in either maternal or cord blood with DSM-IV–oriented Anxiety Problems. The results were materially unchanged following adjustment for postnatal exposures to PAH or ETS. Parameter estimates and CIs for all variables included in final models for ambient PAH, maternal adducts, and cord adducts are provided in Supplemental Material Tables 1, 2, and 3 respectively (http://dx.doi.org/10.1289/ehp.1104315).

## Discussion

This is the first report of an association between child behavioral problems and two complementary measures specific to prenatal PAH exposure: prenatally monitored air concentrations of PAH and a PAH-specific biomarker of exposure (BaP–DNA adducts) in cord blood. The finding of significant associations between two complementary measures of prenatal exposure to PAH and indicators of both Anxious/Depressed and Attention Problems in children is consistent with prior experimental research and with our previous reports indicating that fetal exposure to PAH is associated with impaired cognitive development of children in the cohort (Perera et al. 2006, 2009a). However, the results with the PAH (BaP)–specific adducts measured here by HPLC in cord and maternal blood differ somewhat from those reported by us previously using a broader spectrum of adducts measured in cord blood by ^32^P-postlabeling (Perera et al. 2011). Although the associations with symptoms of attention problems were similar using all three measures (monitored PAH and the two different types of adducts), the broader spectrum of adducts measured by ^32^P-postlabeling was not significantly associated with symptoms of anxiety/depression at 7 years of age; it was, however, significantly and positively associated with symptoms of anxiety/depression at 5 years. This divergence is not unexpected given that the ^32^P-postlabeling method detects adducts formed by a range of hydrophobic aromatic hydrocarbons in addition to PAH, such as nitro-aromatic compounds (e.g., 3-nitrobenzanthrone) (Arlt et al. 2001) and heterocyclic amines (e.g., 4-aminobiphenyl) (Munnia et al. 2007). In contrast, the HPLC method detects the adducts formed by BaP. BaP is considered a representative PAH and in our study was highly correlated with the other 7 genotoxic PAH measured in prenatal air (*r* = 0.80–0.96, *p* = 0.001 except for dibenz[*a,h*]anthracene, *r* = 0.53, *p* < 0.001).

Although in the logistic model only monitored PAH was significantly associated with DSM-IV–oriented Anxiety Problems, most of the other associations between symptom scores and measured PAH are consistent. It is likely that PAH are operating through mechanisms in addition to direct genotoxicity evidenced by DNA adduct formation. In fact, a number of pathways have been suggested including endocrine disruption (Archibong et al. 2002; Bui et al. 1986; Takeda et al. 2004), binding to receptors for placental growth factors resulting in decreased exchange of oxygen and nutrients (Dejmek et al. 2000), binding to the human Ah receptor to induce P450 enzymes (Manchester et al. 1987), DNA damage resulting in activation of apoptotic pathways (Meyn 1995; Nicol et al. 1995; Wood and Youle 1995), oxidative stress due to inhibition of the brain antioxidant scavenging system (Saunders et al. 2006), or epigenetic alterations affecting gene expression (Perera and Herbstman 2011; Wilson and Jones 1983). Fetal BaP exposure also influenced the expression of nuclear transcription factors that mediate the onset of neuronal cell differentiation, suggesting that there may be widespread effects of this agent in the developing brain, ultimately contributing to neurobehavioral impairment (Hood et al. 2000). Although the exposures/doses in the animal studies are higher than those in the NYC cohort, a number of laboratory studies have observed depression-like symptoms and impaired memory in experimental animals exposed gestationally to PAH at doses below those causing overt toxicologic effects (Saunders et al. 2002; Wormley et al. 2004).

The children in the CCCEH cohort are being followed to 12 years of age; therefore subsequent testing will provide a picture of the longer-term developmental outcomes of children in the cohort. The strengths of the study include the fact that we were able to account for a number of factors other than PAH exposure that are known to affect child neurobehavioral development, drawing on individual prenatal exposure data from personal monitoring, biomarker data, and extensive medical record and questionnaire data. We were also able to confirm our findings from prenatal PAH monitoring using BaP–DNA adducts as our dosimeter.

We acknowledge a number of limitations of this epidemiologic study. First, unmeasured factors such as other pollutants and stress may have contributed to residual confounding. Further, a single 48-hr prenatal monitoring during the second or third trimester was used as a basis for estimating exposure. However, this single personal air measurement has been associated with adverse health and developmental outcomes in two cohorts (Choi et al. 2006; Edwards et al. 2010; Perera et al. 2003, 2006, 2009a) and has been correlated with indoor PAH concentrations monitored over a 6-week period as well as with indoor and outdoor PAH concentrations monitored during the same 48-hr time period (Rundle et al. 2012). We therefore consider the single monitoring time point to be a useful indicator of prenatal exposure to PAH via inhalation. In addition, other studies have found that ETS exposure has been associated with behavioral problems (Fergusson et al. 1993; Weitzman et al. 1992). Although we have adjusted for the possible confounding effects of ETS, there is always the possibility that some residual confounding remains, possibly due to measurement error or the shared variance between ETS and PAH measures. Because cord blood cotinine was available only for a subset of participants, we used self-reported ETS exposure as a measure of passive smoking. However, in the smaller sample with cotinine measurements in cord blood, results were similar. Finally, children born severely preterm would not have been included in our analysis because PAH monitoring was carried out in the third trimester, and we excluded active smokers, illicit drug users, and women with preexisting disease. Our findings may therefore not be generalizable to more at-risk populations.

## Conclusion

This study provides evidence that prenatal exposure to environmental PAH at levels encountered in the air of New York City may influence child behavior. The results suggest an adverse impact of prenatal PAH exposure on child behavior that could impact cognitive development and ability to learn. Anxiety, depression, and attention problems, which were associated with PAH exposure and BaP–DNA adducts in our study population, have been shown to affect subsequent academic performance (Emslie 2008; Wood 2006). PAH are widespread in urban environments worldwide largely as a result of fossil fuel combustion. Fortunately, it is possible to reduce airborne PAH concentrations using currently available pollution controls, greater energy efficiency, the use of alternative energy sources, and regulatory intervention to remove highly polluting sources.

## Supplemental Material

(160 KB) PDF
