# Supplementary material for: Prenatal Polycyclic Aromatic Hydrocarbon (PAH) Exposure and Child Behavior at Age 6–7 Years
Source: Environ Health Perspect. 2012 Mar 14;120(6):921–6. doi: 10.1289/ehp.1104315 (PMC3385432; doi:10.1289/ehp.1104315)
Supplement: (160 KB) PDF [file ehp.1104315.s001.pdf]

Supplemental material

Prenatal Polycyclic Aromatic Hydrocarbon (PAH) Exposure and Child Behavior at age 6-7

Frederica P. Perera, Deliang Tang, Shuang Wang, Julia Vishnevetsky, Bingzhi Zhang, Diurka Diaz, David Camann, Virginia Rauh

Contents

Supplemental Material, Table 1. Estimated effects of all variables in the main model with PAH as the main predictor....3

Supplemental Material, Table 2. Estimated effects of all variables in the main model with maternal PAH (B[a]P)-DNA adducts as the main predictor....4

Supplemental Material, Table 3. Estimated effects of all variables in the main model with cord PAH (B[a]P)-DNA adducts as the main predictor....5

Supplemental Table 4. Analysis adjusting for age 3 urinary PAH metabolites....6

Supplemental Material, Table 1. Estimated effects of all variables in the main model with PAH as the main predictor

Variable	Anxious /Depressed				Attention Problems				DSM oriented Anxiety Problem		DSM oriented ADHD Problem	
	Poisson Raw		Logistic Dichotomized T		Poisson Raw		Logistic Dichotomized T		Logistic Model		Logistic Model	
	β (95% CI)	p-value	β (95% CI)	p-value	β (95% CI)	p-value	β (95% CI)	p-value	β (95% CI)	p-value	β (95% CI)	p-value
Intercept	-2.69 (-8.30, 2.92)	0.347	-11.68 (-52.95, 29.60)	0.579	-1.20 (-5.89, 3.49)	0.616	-15.40 (-50.99, 20.19)	0.396	-15.57 (-48.83, 17.69)	0.359	4.96 (-29.95, 39.88)	0.781
PAH (high/low)	0.37 (0.20, 0.54)	<.0001	2.19 (0.53, 3.84)	0.01	0.24 (0.10, 0.39)	0.001	1.33 (0.13, 2.54)	0.030	1.52 (0.38, 2.66)	0.009	0.83 (-0.24, 1.90)	0.129
Prenatal ETS	-0.01 (-0.18, 0.16)	0.929	0.63 (-0.58, 1.83)	0.308	0.04 (-0.10, 0.19)	0.574	-0.33 (-1.49, 0.83)	0.574	0.05 (-0.95, 1.06)	0.917	0.77 (-0.23, 1.78)	0.132
Gender	-0.01 (-0.17, 0.15)	0.885	-0.44 (-1.56, 0.68)	0.446	-0.40 (-0.54, -0.26)	<.0001	-0.72 (-1.81, 0.37)	0.198	-0.72 (-1.67, 0.23)	0.136	-0.18 (-1.19, 0.84)	0.734
Gestational age	-0.05 (-0.10, 0.00)	0.042	-0.35 (-0.67, -0.03)	0.032	-0.05 (-0.09, -0.01)	0.017	-0.07 (-0.38, 0.23)	0.648	-0.26 (-0.53, 0.00)	0.049	-0.14 (-0.42, 0.14)	0.317
Maternal IQ	-0.01 (-0.02, 0.00)	0.167	-0.01 (-0.08, 0.05)	0.727	0.00 (-0.01, 0.01)	0.862	-0.02 (-0.08, 0.05)	0.587	0.00 (-0.06, 0.05)	0.933	0.00 (-0.06, 0.06)	0.969
HOME Inventory	-0.01 (-0.03, 0.00)	0.105	-0.09 (-0.19, 0.02)	0.095	0.01 (0.00, 0.03)	0.097	0.06 (-0.05, 0.17)	0.279	-0.07 (-0.16, 0.02)	0.126	-0.02 (-0.11, 0.08)	0.715
Maternal education	0.00 (-0.04, 0.04)	0.965	-0.03 (-0.32, 0.26)	0.845	-0.05 (-0.09, -0.01)	0.007	0.02 (-0.25, 0.28)	0.911	-0.06 (-0.28, 0.17)	0.612	-0.11 (-0.36, 0.15)	0.406
Ethnicity	-0.28 (-0.46, -0.10)	0.002	-0.88 (-2.20, 0.43)	0.188	0.02 (-0.13, 0.18)	0.75	0.00 (-1.17, 1.16)	0.994	-0.69 (-1.78, 0.41)	0.218	-0.03 (-1.11, 1.05)	0.962
Prenatal demoralization	0.17 (0.05, 0.30)	0.008	0.06 (-0.89, 1.01)	0.900	0.24 (0.12, 0.35)	<.0001	1.17 (0.30, 2.04)	0.009	0.62 (-0.14, 1.38)	0.112	0.95 (0.17, 1.72)	0.016
Age at assessment	0.07 (0.01, 0.14)	0.029	0.31 (-0.19, 0.81)	0.229	0.05 (0.00, 0.11)	0.057	0.14 (-0.28, 0.57)	0.503	0.31 (-0.09, 0.72)	0.127	-0.02 (-0.42, 0.39)	0.934
Heating season	-0.17 (-0.34, 0.01)	0.06	-0.90 (-2.13, 0.32)	0.149	-0.23 (-0.38, -0.09)	0.002	-1.21 (-2.39, -0.03)	0.044	-0.71 (-1.75, 0.34)	0.185	-0.91 (-1.97, 0.15)	0.094

Coding: Ethnicity was coded as 1=African American, 0=Dominican;

Supplemental Material, Table 2. Estimated effects of all variables in the main model with maternal PAH (B[a]P)-DNA adducts as the main predictor

Variable	Anxious /Depressed				Attention Problems				DSM oriented Anxiety Problem		DSM oriented ADHD Problem	
	Poisson Raw		Logistic Dichotomized T		Poisson Raw		Logistic Dichotomized T		Logistic Model		Logistic Model	
	β (95% CI)	p-value	β (95% CI)	p-value	β (95% CI)	p-value	β (95% CI)	p-value	β (95% CI)	p-value	β (95% CI)	p-value
Intercept	-3.84 (-9.74, 2.05)	0.201	-18.69 (-65.01, 27.63)	0.429	-0.05 (-4.83, 4.73)	0.984	-17.16 (-53.53, 19.21)	0.355	-19.80 (-54.94, 15.34)	0.269	6.29 (-29.35, 41.93)	0.729
Maternal adducts	0.21 (0.03, 0.38)	0.019	0.35 (-0.97, 1.68)	0.603	0.23 (0.08, 0.37)	0.003	0.81 (-0.30, 1.91)	0.153	0.78 (-0.24, 1.80)	0.133	0.61 (-0.41, 1.63)	0.243
Prenatal ETS	0.01 (-0.17, 0.19)	0.914	1.19 (-0.21, 2.59)	0.097	0.04 (-0.12, 0.19)	0.643	-0.04 (-1.24, 1.16)	0.947	0.27 (-0.81, 1.35)	0.622	1.07 (0.01, 2.14)	0.048
Gender	0.12 (-0.05, 0.30)	0.175	0.23 (-1.12, 1.58)	0.736	-0.35 (-0.50, -0.21)	<.0001	-0.57 (-1.70, 0.56)	0.319	-0.28 (-1.32, 0.76)	0.598	-0.09 (-1.16, 0.98)	0.873
Gestational age	-0.04 (-0.09, 0.02)	0.182	-0.28 (-0.65, 0.09)	0.132	-0.03 (-0.07, 0.02)	0.239	-0.07 (-0.38, 0.25)	0.676	-0.21 (-0.49, 0.08)	0.154	-0.13 (-0.42, 0.16)	0.393
Maternal IQ	-0.01 (-0.02, 0.00)	0.225	0.01 (-0.08, 0.09)	0.903	0.00 (-0.01, 0.01)	0.523	0.00 (-0.07, 0.07)	0.987	0.01 (-0.06, 0.07)	0.809	0.02 (-0.05, 0.09)	0.533
HOME Inventory	-0.01 (-0.03, 0.01)	0.253	-0.11 (-0.23, 0.01)	0.063	0.00 (-0.01, 0.02)	0.528	0.06 (-0.05, 0.17)	0.268	-0.07 (-0.16, 0.02)	0.139	-0.02 (-0.12, 0.07)	0.617
Maternal education	0.02 (-0.02, 0.07)	0.271	0.16 (-0.21, 0.52)	0.397	-0.04 (-0.08, 0.00)	0.039	0.05 (-0.24, 0.34)	0.730	0.02 (-0.22, 0.27)	0.857	-0.08 (-0.35, 0.19)	0.551
Ethnicity	-0.33 (-0.53, -0.13)	0.001	-1.22 (-2.81, 0.36)	0.131	0.02 (-0.14, 0.19)	0.778	-0.33 (-1.57, 0.90)	0.596	-0.90 (-2.13, 0.32)	0.148	-0.23 (-1.37, 0.92)	0.699
Prenatal demoralization	0.07 (-0.07, 0.21)	0.330	-0.43 (-1.58, 0.73)	0.468	0.15 (0.03, 0.27)	0.013	0.65 (-0.22, 1.52)	0.145	0.34 (-0.47, 1.14)	0.415	0.57 (-0.23, 1.36)	0.161
Age at assessment	0.08 (0.01, 0.15)	0.031	0.36 (-0.21, 0.92)	0.215	0.03 (-0.03, 0.09)	0.294	0.16 (-0.27, 0.59)	0.465	0.33 (-0.10, 0.76)	0.128	-0.04 (-0.45, 0.37)	0.848
Heating season	-0.10 (-0.28, 0.08)	0.290	-0.77 (-2.17, 0.64)	0.286	-0.17 (-0.32, -0.02)	0.031	-0.57 (-1.76, 0.61)	0.345	-0.36 (-1.47, 0.74)	0.521	-0.45 (-1.53, 0.63)	0.416

Supplemental Material, Table 3. Estimated effects of all variables in the main model with cord PAH (B[a]P)-DNA adducts as the main predictor

Variable	Anxious /Depressed				Attention Problems				DSM oriented Anxiety Problem		DSM oriented ADHD Problem	
	Poisson Raw		Logistic Dichotomized T		Poisson Raw		Logistic Dichotomized T		Logistic Model		Logistic Model	
	β (95% CI)	p-value	β (95% CI)	p-value	β (95% CI)	p-value	β (95% CI)	p-value	β (95% CI)	p-value	β (95% CI)	p-value
Intercept	-4.81 (-11.83, 2.21)	0.179	-6.93 (-54.60, 40.74)	0.776	-0.27 (-6.09, 5.54)	0.927	-6.06 (-44.31, 32.18)	0.756	-5.97 (-45.94, 33.99)	0.770	-4.19 (-41.94, 33.57)	0.828
Cord adducts	0.38 (0.18, 0.58)	0.000	0.94 (-0.37, 2.24)	0.159	0.28 (0.10, 0.46)	0.002	1.40 (-0.01, 2.81)	0.051	0.93 (-0.18, 2.03)	0.101	0.97 (-0.39, 2.33)	0.161
Prenatal ETS	-0.03 (-0.24, 0.19)	0.820	0.55 (-0.77, 1.88)	0.413	0.14 (-0.04, 0.33)	0.138	-0.25 (-1.72, 1.21)	0.735	-0.13 (-1.34, 1.07)	0.827	0.56 (-0.78, 1.89)	0.414
Gender	-0.19 (-0.39, 0.01)	0.063	-0.74 (-2.03, 0.55)	0.260	-0.42 (-0.59, -0.25)	<.0001	-0.58 (-1.90, 0.73)	0.383	-1.00 (-2.12, 0.12)	0.079	-0.75 (-2.08, 0.59)	0.271
Gestational age	-0.04 (-0.10, 0.02)	0.224	-0.36 (-0.73, 0.02)	0.062	-0.05 (-0.10, 0.01)	0.076	-0.05 (-0.46, 0.36)	0.804	-0.33 (-0.65, -0.01)	0.043	0.05 (-0.39, 0.49)	0.828
Maternal IQ	-0.01 (-0.03, 0.00)	0.049	-0.01 (-0.09, 0.07)	0.787	0.00 (-0.01, 0.01)	0.637	0.00 (-0.08, 0.09)	0.936	0.01 (-0.06, 0.07)	0.867	0.01 (-0.07, 0.09)	0.817
HOME Inventory	-0.02 (-0.04, 0.00)	0.060	-0.05 (-0.16, 0.06)	0.378	0.01 (-0.01, 0.03)	0.268	0.08 (-0.06, 0.22)	0.247	-0.07 (-0.16, 0.03)	0.193	0.06 (-0.07, 0.20)	0.357
Maternal education	0.04 (-0.01, 0.09)	0.079	0.10 (-0.25, 0.45)	0.570	-0.01 (-0.05, 0.04)	0.722	0.16 (-0.21, 0.53)	0.405	0.06 (-0.21, 0.32)	0.679	0.04 (-0.32, 0.40)	0.818
Ethnicity	-0.25 (-0.49, -0.01)	0.040	-0.73 (-2.26, 0.80)	0.351	0.04 (-0.16, 0.24)	0.666	0.03 (-1.46, 1.52)	0.971	-0.74 (-2.06, 0.57)	0.269	0.23 (-1.24, 1.70)	0.759
Prenatal demoralization	0.19 (0.03, 0.35)	0.019	-0.12 (-1.21, 0.98)	0.835	0.20 (0.06, 0.35)	0.007	0.38 (-0.72, 1.47)	0.498	0.50 (-0.40, 1.40)	0.278	1.06 (0.02, 2.10)	0.046
Age at assessment	0.09 (0.01, 0.17)	0.027	0.23 (-0.33, 0.80)	0.419	0.04 (-0.03, 0.10)	0.261	0.00 (-0.43, 0.42)	0.985	0.22 (-0.25, 0.69)	0.358	-0.06 (-0.47, 0.34)	0.765
Heating season	0.03 (-0.18, 0.25)	0.770	0.28 (-1.15, 1.71)	0.697	-0.22 (-0.40, -0.03)	0.021	-0.55 (-1.93, 0.83)	0.433	0.04 (-1.16, 1.24)	0.948	-0.32 (-1.70, 1.06)	0.654

Supplemental Table 4. Analysis adjusting for age 3 urinary PAH metabolites.

	Anxious Depressed			Attention		
Variable	Beta	95% CI	P-value	Beta	95% CI	P-value
Intercept	-0.25	(-7.68, 7.19)	0.948	-3.58	(-9.91, 2.76)	0.268
PAH (high/low)	0.54	(0.34, 0.74)	<.0001	0.32	(0.15, 0.50)	0.000
Prenatal ETS	0.03	(-0.19, 0.24)	0.806	-0.06	(-0.24, 0.12)	0.501
Gender	-0.01	(-0.20, 0.18)	0.947	-0.38	(-0.55, -0.21)	<.0001
Gestational age	-0.09	(-0.15, -0.04)	0.001	-0.04	(-0.09, 0.01)	0.124
Maternal IQ	-0.01	(-0.02, 0.00)	0.104	-0.01	(-0.02, 0.00)	0.238
HOME Inventory	-0.02	(-0.03, 0.00)	0.058	0.00	(-0.01, 0.02)	0.653
Maternal education	0.03	(-0.02, 0.09)	0.251	-0.01	(-0.06, 0.03)	0.581
Ethnicity	-0.27	(-0.49, -0.04)	0.019	0.08	(-0.11, 0.28)	0.385
Prenatal demoralization	0.16	(0.00, 0.31)	0.049	0.18	(0.04, 0.32)	0.012
Age at assessment	0.06	(-0.03, 0.14)	0.205	0.07	(-0.01, 0.14)	0.071
Heating season	-0.29	(-0.50, -0.09)	0.005	-0.37	(-0.55, -0.20)	<.0001
PAH metabolites	0.05	(-0.06, 0.15)	0.384	0.07	(-0.01, 0.16)	0.099
